# Effect of dendritic organ ligation on striped eel catfish *Plotosus lineatus* osmoregulation

**DOI:** 10.1371/journal.pone.0206206

**Published:** 2018-10-23

**Authors:** Salman Malakpour Kolbadinezhad, João Coimbra, Jonathan M. Wilson

**Affiliations:** 1 Centro Interdisciplinar de Investigação Marinha e Ambiental, Universidade do Porto, Matosinhos, Portugal; 2 Instituto de Ciências Biomédicas de Abel Salazar, Universidade do Porto, Porto, Portugal; 3 Department of Biology, Wilfrid Laurier University, Waterloo, Canada; Helmholtz-Zentrum fur Ozeanforschung Kiel, GERMANY

## Abstract

Unique amongst the teleost, Plotosidae catfish possess a dendritic organ (DO) as a purported salt secreting organ, whereas other marine teleosts rely on their gill ionocytes for active NaCl excretion. To address the role of the DO in ionregulation, ligation experiments were conducted in brackish water (BW) 3‰ and seawater (SW) 34‰ acclimated *Plotosus lineatus* and compared to sham operated fish. Ligation in SW resulted in an osmoregulatory impairment in blood (elevated ions and hematocrit) and muscle (dehydration). However, SW ligation did not elicit compensatory changes in gill or kidney Na^+^/K^+^-ATPase (NKA) activity and/or protein expression while a decrease in anterior intestine and increased in posterior intestine were observed but this was not reflected at the protein level. Following ligation in SW, protein levels of carbonic anhydrase (CA) and V-ATPase B subunit (VHAB) were higher in kidney but either lower (CA) or unchanged (VHAB) in other tissues. Taken together, the osmotic disturbance in ligated SW fish indicates the central role of the DO in salt secretion and the absence of a compensatory response from the gill.

## Introduction

The gill is the central ionregulatory organ in teleost fishes with supporting roles played by the kidney and intestine [1, 2]. Branchial mitochondrion-rich cells (MRCs) or ionocytes, are well characterized [1, 3, 4] and function in freshwater and saltwater to actively take up and excrete monovalent ions (Na^+^, K^+^, and Cl^-^) respectively, to maintain plasma osmolality [1, 3]. In marine environments, teleost fishes are faced with passive salt uptake and dehydration, which require active compensation for homeostasis [2]. Branchial ionocytes use secondary active transport for transcellular Cl^-^ secretion [1]. Basolateral Na^+^/K^+^-ATPase (NKA) creates a favorable Na^+^ gradient for Cl^-^ entry into the cell via the Na^+^: K^+^:2Cl^-^ cotransporter (NKCC1) from the extracellular fluid against chloride’s electrochemical gradient. Cl^-^ exits the cell apically down its electrochemical gradient via a CFTR Cl^-^ channel. Na^+^, on the other hand, takes a paracellular path through leaky tight junctions with neighboring accessory cells. Marine teleosts drink continuously at approximately 1–5 ml per kg per h to compensate for osmotic water losses [2]. The imbibed seawater is first desalinated by the esophagus, which is highly permeable to Na^+^ and Cl^-^ but not water. The water that reaches the intestine is only slightly hyperomotic to plasma, and solute-linked water transport is used to drag water across the epithelium by creating regions of localized hypertonicity in the lateral intercellular spaces [5]. The majority of water uptake occurs in the anterior and mid regions of the intestine [6, 7]. This process is driven by NaCl transport involving apical Na^+^: K^+^: 2Cl^-^ cotransporter-2 (NKCC2), Na^+^: Cl^-^ cotransporter (NCC) and sodium linked glucose transporter-1 (SGLT-1) to facilitate apical ion movements and the basolateral NKA that creates favourable electrochemical conditions resulting in the absorption of 39–85% of imbibed water [7]. As water and monovalent ions are absorbed, divalent ions (Ca^2+^, Mg^2+^ and SO_4_^2-^) potentially increase in concentration in the intestinal lumen, which can impede the osmotic movement of water. This potential problem is addressed by the secretion of bicarbonate via an apical Cl^-^/HCO_3_^-^ exchange that results in alkaline luminal pH levels (up to pH 9) and consequently the precipitation of divalent cations as CaCO_3_ and MgCO_3_ accounting for 20–60% of the total excreted [6, 7]. The apical Cl^-^/HCO_3_^-^ exchanger has been identified as a *slc26a6* [8]. Cytosolic carbonic anhydrase (CA) plays an important role in providing an intracellular pool of HCO_3_^-^ for the exchanger [6]. This system may be augmented in the posterior intestine by an apical H^+^-ATPase that titrates some of the excreted HCO_3_^-^ to allow for continued Cl^-^ uptake via Cl^-^/HCO_3_^-^ exchange to enhance water uptake [6, 9]). The CFTR Cl^-^ channel has a basolateral localization to facilitate Cl^-^ exit across the basolateral membrane although it may also be important in salt and fluid secretion in marine teleosts when expressed apically ([10]. The kidney in marine teleosts generally produces small volumes of iso-osmotic urine that is importantly the main route for the excretion of toxic divalent ions (Ca^2+^, Mg^2+^ and SO_4_^2-^) (reviewed by [2, 11].

Freshwater fishes are faced with the opposite challenges of osmotic water gain and passive ion losses across their body surfaces [2]. The gills are the primary site for active ion uptake but the molecular mechanisms are variable and species dependent [1, 3]. Mechanisms of Na^+^ uptake include the apical NCC, NHE (Na^+^/H^+^ exchangers 2,3), and ASIC (acid sensing ion channels) Na^+^ channel coupled to the vacuolar type H^+^ ATPase (VHA), while Cl^-^ uptake involves a Cl^-^/HCO_3_^-^ exchanger (Slc26a6) [3, 12]. The basolateral NKA is also central to driving ion uptake directly or indirectly through the sodium motive force [1]. In addition, cytosolic carbonic anhydrase (Ca17; [13]) plays an important role in providing an intracellular pool of H^+^ and HCO_3_^-^ for some of these transporters (e.g. NHE and Slc26a6) from the catalyzed dehydration of CO_2_ [14]. Freshwater fishes do not actively drink but the diet is an important source of ions, which are absorbed by the gut [15]. The freshwater fish kidney takes on a prominent role in the excretion of excess osmotically gained water through the production of very dilute urine through the combination of a high glomerular filtration rate and active ion reabsorption [11]. This ion reabsorption is driven largely by the basolateral NKA [2].

In contrast to other teleosts, in the Plotosidae marine catfishes [16] there is ultrastructural [16, 17], histochemical [18] and physiological [19] evidence suggesting that the unique Plotosidae dendritic organ (DO) is responsible for salt excretion. The DO was first described by Bloch [20] followed by Brock [21] and Hirota [22]. It is a well vascularized, small fleshy external branching organ situated very close, to the urogenital papilla on the ventral surface of the fish. Kowarsky [19] found a significantly reduced survival of DO ligated catfish *Cnidoglanism acrocephalus* and increase in plasma Na^+^ concentrations in fish at hyperosmotic salinities. High Cl^-^ levels have been demonstrated in DO parenchymal cells, similar to gill chloride cells [23]. More recently, it has been demonstrated that the DO parenchymal cells express high levels of NKA and NKCC1 and have an apical CFTR Cl^-^ channel consistent with the secondary activity chloride secretion mechanism seen in other salt secreting epithelia in vertebrates including the gills of typical marine teleost fishes [24].

In this study, we test the hypothesis that the DO is central to hypo-osmoregulation in *Plotosus lineatus* by DO ‘knock out’ through ligation. We also address whether the gill, which is the central ionoregulation organ in teleost fishes, and other osmoregulatory organs (kidney and intestine) show compensatory responses to DO ligation. To this end, we measure a suite of osmoregulatory end points (plasma and muscle ion concentrations and muscle water levels) and ion transporter expression levels in seawater and brackish water acclimated, DO ligated and sham ligated *P*. *lineatus*. Ion transporter (NKA, VHA, NKCC1/2 NCC) expression was assessed primarily at the protein level through a combination of immunohistochemistry (localization) and Western blotting (organ level expression) complemented by qPCR and transcript level expression (NKA: *atp1a1*; *cftr*; *ca17*; *slc26a6*). In addition, NKA activity was also measured. It should be noted that antibodies against Slc26a6 were not crossreactive [8] and bands were not detected by Western blotting for either Cftr, or NKCC1/2 NCC. Osmotic shock has been shown to result in an organ specific cellular stress response in fishes as indicated by elevated Hsp70 (heat shock protein 70) levels [24, 25]. Therefore, we also assessed if ligation resulted in an osmotic stress induced induction of Hsp 70 expression (by western blotting).

## Material and methods

### Animals

The striped eel catfish *Plotosus lineatus* (~8–13 g) were purchased from TMC Portugal and transported to the Laboratory of Molecular Physiology CIIMAR (Porto). Prior to the start of the experiments fish were acclimated to a 100 L tank with seawater (SW) 34‰, natural photoperiod and biological and mechanical filtration with UV sterilization (HW-303B, Sun Sun, China) for three weeks prior to the start of the experiment to avoid any confounding effects of handling stress on osmoregulation [26]. Seawater was made up using Instant Ocean salt. Fish were fed twice daily with diced fish fillets during this period but not fed 4 days before samplings. Periodic 20% water changes were also made. Water temperature (range at 26–28°C), pH (range pH 7.7–7.9), salinity and dissolved oxygen (340i multimetric probe, WTW Measurement Systems Inc. Weilheim, Germany) as well as fish behaviour were monitored daily. Fish were used according to the Portuguese Animal Welfare Law (Decreto-Lei no.197/96) and animal protocols were approved by the Interdisciplinar Centre for Marine and Environmental Research (CIIMAR)-University of Porto and Direction General Veterinarian (DGV).

### Salinity acclimation

Two salinity levels were investigated: brackish water (BW) 3‰, and seawater (SW) 34‰. Initially, individuals were transferred to a 22 L tank (small tank) with mechanical and biological filtration and UV sterilization. The salinity was changed by removing water and adding an appropriate volume of dechlorinated tap water in a stepwise fashion to decrease the salinity from 34‰ to 3‰ by 5‰ per day. Fish were kept in the same tank to minimize disturbances from handling. A water change of the SW-control group was also conducted in order to standardize fish stress at each salinity change between the different groups. Fish were checked twice daily and moribund fish were removed from the experimental tanks. These fish have lost equilibrium and were euthanized with an overdose of MS222 (Pharmaq UK). Fish were acclimated for at least two weeks at the final salinities.

### Ligation experiment

Fish were fasted for four days and then anaesthetized with 1:10 000 MS222. The DO was ligated using suture thread in BW and SW acclimated fish as described by Kowarsky [19] in *C*. *macrocephalus*. In preliminary experiments, a group of fish acclimated to hypersaline water (60‰) were ligated but did not survive and ligation experiments were not pursued further with these fish. Control fish were anaesthetized and sham ligated. Fish that did not recover or were unable to maintain equilibrium during the experiments were euthanized with an overdose of MS-222 (1:5000). The data on sham ligated fish has been published in Malakpour Kolbadinezhad et al. [24].

### Sampling

*P*. *lineatus* were anaesthetized with an overdose of MS-222 (1:5000, pH 7.5 adjusted with NaHCO_3_) 48h after ligation or sham ligation. Blood was collected using a heparinized capillary tube following caudal transection, centrifuged at 13000xg for 5min (Heraeus Pico 17 Centrifuge, Thermo Scientific) at room temperature. The hematocrit (Hct) was measured in duplicate (nearest mm) then converted to percentage of total blood volume. Gill, kidney, anterior and posterior intestine were excised and either (1) directly snap frozen in liquid nitrogen or (2) immersed in SEI buffer [sucrose (150 mM), EDTA (10 mM), imidazole (50 mM), pH 7·3] and then snap frozen and stored at -80°C. Blood sampling was done in additional sets of six individuals but the body cavities were opened by ventral incision and the whole carcasses were immersion fixed in 10% neutral buffered formalin (NBF 10%) for 24h and stored in 70% ethanol at 4°C for immunohistological analyses.

### Plasma and muscle analyses

To determine muscle water content (MWC), approximately one gram of muscle was collected and dried to constant mass at 60°C. For ion quantification, dried muscle was digested in five volumes of nitric acid (65%) for three days at room temperature until completely dissolved. Na^+^ and K^+^ concentrations were quantified in plasma and muscle samples by flame photometery (PinAAcle 900T Atomic Absorption Spectrophotometer; Perkin Elmer Waltham MA). Chloride concentrations of plasma were measured by the mercuric thiocyanate reaction method [27]. Plasma osmolality was determined by freezing-point depression (Melting Point Osmometer, N 961003, Roebling Co. Germany) and reported as mOsm kg^−1^ [28].

### Measurement of Na^+^/K^+^-ATPase activity

NKA activity was measured according to the microassay protocol of McCormick [29] with some modifications. The tissues were thawed and then homogenized at 5800 RPM for 2x15s (Precellys 24 homogenizer Bertin Technologies, France) in SEI (250 mM sucrose, 10 mM EDTA, 50 mM imidazole pH 7.3) buffer containing 0·1% deoxycholic acid and centrifuged at 15,000xg (5 minutes at 4 °C) to remove large debris. Ten μl of supernatant was added to 200μl of assay mixture while the assay were run in two sets of duplicate, one set containing the assay mixture and the other assay mixture plus the specific NKA activity inhibitor, ouabain (1 mM, Sigma–Aldrich Chemical Co.). ATPase activity was measured with a temperature controlled plate reader (Powerwave 340; Biotek, Winooski, VT) at 340nm for 10–20 min at 25°C. Total protein concentrations were determined with a bovine serum albumin (BSA) standard by Bradford [30] dye binding assay at 600nm. The results are expressed as μmoles ADP ·mg^−1^ protein· h^−1^.

### Western blotting

Tissue samples were homogenized with a Precellys 24 homogenizer in 50mM imidazole buffer pH 7.5 at 5800 RPM for 2x15s and immediately centrifuged at 15,000g for 5 min at 4°C. The supernatant was mixed with an equal volume of 2x Laemmli’s buffer [31], heated (10 min at 70°C) and then stored at 4°C. Total protein was measured in leftover supernatant using the Bradford method. Sample protein concentrations were adjusted to 1 μg μl^-1^ using 1x Laemmli’s buffer. Ten to 20μg of sample were separated on 10%T (total percent concentration (w:v) of acrylamide) mini vertical polyacrylamide gels with 4%T stacking gels (BioRad Hercules CA USA). Gels were then equilibrated in transfer buffer and bands were transferred to nitrocelulose membranes (GE Healthcare Amersham Hybond ECL) using a BioRad SD-transblot. Membranes were then rinsed in 0.05% Tween-20 in Tris Buffered Saline, pH 7.5 (TTBS) and blocked with 10% powdered skim milk in TTBS for 1h. Blots were probed with heterologous antibodies to NKA α subunit (rabbit αR1 antibody; [32]), NKCC1 (mouse monoclonal T4 antibody; DSHB, U.Iowa USA; [32]), carbonic anhydrase (bovine CA2 antibody; abcam, Cambridge UK, [33]), VHA B subunit (rabbit polyclonal B2 antibody; [32]), Slc26a6a and Slc26a6b (rabbit polyclonals; [8]) and heat shock protein 70 (Hsp70; monoclonal BRM-22 antibody; Sigma Aldrich), overnight at room temperature in 50 ml centrifuge tubes on a rotisserie (LabQuake2; Thermo Fisher Sci.). Membranes were then rinsed with TTBS and incubated for 1 hour with either a goat anti-rabbit or anti-mouse IgG secondary antibodies conjugated to horseradish peroxidase diluted in TTBS (1:50000). Membranes were rinsed a final time with TTBS and signal was obtained by enhanced chemiluminescence (ECL) using Millipore Immobilon Western chemiluminescent HRP substrate (Millipore Corporation, USA). Images were acquired using a Fujifilm LAS-4000 mini and image reader software LAS-4000 version.2.0. The intensity of the band signals were quantified using Multi Gauge v3.1 (Fujifilm, JPN).

### Immunohistochemistry

Paraffin serial sections (5 μm) were cut and collected onto APS (3- aminopropyltriethoxysilane; Sigma Aldrich) coated slides [34], completely dried, and then dewaxed and rehydrated. Antigen retrieval was performed on some sections [35] by pretreatment with 0.05% citraconic anhydride (pH 7.3) for 30min at 98°C [36] followed by 1% sodium dodecyl sulfate (SDS) in PBS [37] at room temperature for 5 min. In between retrieval steps, sections were rinsed with MilliQ water, air-dried and circled with a liquid hydrophobic blocker (Super PAP pen Sigma Aldrich). Prior to antibody incubation, sections were blocked with 5% normal goat serum (NGS) in 1% BSA in TPBS (0.05% Tween-20 in Phosphate Buffered Saline, pH 7.4) and then incubated with primary antibodies against the α-subunit of NKA (1:500 αR1), NKCC1 (1:100 T4), carbonic anhydrase (1:200 CA), CFTR (R&D systems) and VHA B-subunit (1:500 B2) diluted in 1%BSA in TPBS for 1-2h at 37°C in a humidity chamber. Sections were then rinsed with TPBS followed by incubation with goat anti-mouse Alexa Fluor 568 and goat anti-rabbit Alexa Fluor 488-conjugated secondary antibodies in (1:500) TPBS for 1h at 37°C. Sections were rinsed in TPBS, stained with DAPI (1:25000) and coverslips mounted with 1:1 glycerol PBS. Sections were viewed on a Leica DM6000B wide field epifluorescence microscope with a digital camera (DFC340FX) using Leica LAS-AF software (Leica Microsystems, Wetzlar, Germany).

### Isolation and quantification of RNA and synthesis of complementary DNA

Total RNA was extracted from frozen gill, kidney and intestine samples using silica-based columns (Aurum Total RNA mini kit, (Bio-Rad). Total RNA concentrations and purity were assessed using a Nanodrop spectrophotometer (Thermo Scientific, USA) and then stored at −80°C. One μg of total RNA was converted to cDNA (iScript cDNA kit; Bio-Rad). Samples were stored at −20°C.

### Gene isolation

Primers designed from the conserved regions of β-actin (*actb*, *Sparus aurata*, [38]), Na^+^/K^+^-ATPase α subunit (*atp1a*, *Anguilla anguilla*, [39]), cystic fibrosis transmembrane conductance regulator [*cftr* (abcc7) *Ictalurus punctatus*, [40]], carbonic anhydrase (*ca17*; *Danio rerio*, [13]), and putative anion transporter Cl^-^/HCO_3_^-^ exchanger (*slc26a6*, *D*. *rerio*, *Tetraodon nigroviridis*, *A*. *anguilla*, *Xenopus laevis*, *Homo sapiens*, [41]) were used for PCR to obtain partial sequence of *P*. *lineatus* orthologs. Nucleotide sequences and amplicon sizes of these primers are shown in S1 Table. PCR amplification products of the correct size were cloned (pGEM-T Easy Vector System, Promega, Madison, WI, USA), sequenced (StabVida, Oeiras, Portugal) and then analyzed for sequence similarity (BLAST, ClustalX). Primer3 [42] was used to design specific *P*. *lineatus* primers and initially tested for specificity by RT-PCR (S2 Table).

### Real-time PCR

The real-time PCR (RT PCR) was performed using BioRad SYBR green supermix with an iQ5 Multicolor Real-Time PCR Detection System (Bio-Rad). The generation of a melt curve for every PCR product (to confirm the specificity of the assays) and preparation of a dilution series to check the efficiency of the reactions has been used. The β-Actin (*bact*), was used as the reference gene. Analysis of the expression levels of the genes of interest was done based on cycle threshold (CT) values using the comparative CT method (2^−ΔΔCT^ method) [43] (S3 Table).

### Statistics

Data are presented as means ± standard deviation (S.D.). Plasma and muscle data were analyzed by one-way analysis of variance (ANOVA) followed by the post hoc Student-Newman-Keuls (SNK) test whereas all other data was analyzed using two-way ANOVA test and SNK (SigmaPlot 11.0 Systat Software, Inc.). Data were log or square root transformed in the case of a failed normality test. Fiducial limit was set at 0.05.

## Results

### Osmoregulatory indicators

During acclimation, there was mortality (50%) in SW acclimation ligated fish (48h) but not in SW sham or either BW group. Attempts at ligation in HSW acclimated fish resulted in 100% mortality so experiments were not pursued further in this group of fishes.

Plasma and muscle osmoregulatory indicators are presented in the Table 1. The SW ligation [(SW-L) 34‰] resulted in higher plasma ion concentrations including Na^+^ and Cl^-^, hematocrit and muscle [Na^+^] (at 24h) and [K^+^] (at 48h). Ligation of BW fish did not result in any differences with BW control fish. However, BW acclimation did lower plasma [Na^+^] and [Cl^-^], and hematocrit. The resulting plasma strong ion ratio was significantly lower in BW fish compared to SW and SW-L fish. Acclimation salinity had no effect on plasma K^+^ or Ca^2+^ concentrations.

**Table 1:** Plasma Na^+^, Cl^-^, K^+^, Ca^2+^concentrations, hematocrit, and strong ion ratio (SIR; Na^+^:Cl^-^) and muscle water content (MWC) and muscle Na^+^ and K^+^ concentrations, and muscle Na^+^/K^+^ ratio of *P*. *lineatus* acclimated to [brackish water (BW), brackish water ligated (BW-L) 3‰, seawater (SW-control), seawater ligated (SW-L) 34‰] salinity). Data are means ± S.D. (n = 7–9). Means within a given parameter across treatment groups which do not share the same letter are significantly different from one another (one-way ANOVA, SNK).

Plasma	BW (3‰)*	BW-L (3‰)	SW-Control (34‰)*	SW-L24h (34‰)	SW-L48h (34‰)
**Na**^**+**^ **(mmol l**^**-1**^**)**	122.6 ± 14.7 ^a^	118.7 ± 10.3 ^a^	148.7 ± 11.70 ^b^		199.2 ± 36.8 ^c^
**Cl**^**-**^ **(mmol l**^**-1**^**)**	127.9 ±12.7 ^a^	117.3 ± 7.8 ^a^	129.5 ± 10.5 ^a^		148.3 ± 10.1 ^b^
**K**^**+**^ **(mmol l**^**-1**^**)**	5.5 ± 0.8	3.2 ± 1.3	5.80 ± 1.0		5.27 ± 2.54
**Ca**^**2+**^ **(mmol l**^**-1**^**)**	2.5 ± 0.5	2.2 ± 0.7	3.9 ± 0.22		3.36 ± 1.20
**Osmolality (mosm kg**^**-1**^**)**	391.2 ± 112.8 ^a^	395.0 ± 67.3 ^a^	374.4 ± 20.6 ^a^	549.3 ± 72.1 ^b^	772.3 ± 165.1 ^c^
**Haematocrit (%)**	14.4± 3.4^a^	13.0 ± 2.3 ^a^	24.5 ± 6.2 ^b^	18.8± 4.2^ab^	33.7 ± 6.9 ^c^
**SIR**	0.9 ± 0.1	1.0 ± 0.1 ^ab^	1.19 ± 0.18^c^		1.21± 0.10 ^bc^
**Muscle**					
**Water content (%)**	85.5 ± 5.1 ^a^	89.7 ± 4.7 ^a^	88.5± 3.3 ^a^	87.5 ± 3.3 ^a^	78.4 ± 5.1 ^b^
**Na**^**+**^ **(mmol kg**^**-1**^**)**	65.7 ± 20.9 ^a^	58.2 ± 19.7 ^a^	67.9± 9.6 ^a^	97.0 ± 21.6 ^b^	74.4 ± 15.8 ^a^
**K**^**+**^ **(mmol kg**^**-1**^**)**	132.0 ± 26.1 ^a^	125.9 ± 29.1 ^a^	144.5 ± 18.6 ^ab^	148.0 ± 39.5 ^ab^	174.8± 26.8 ^b^
**Na**^**+**^**/K**^**+**^ **ratio**	0.6 ± 0.3	0.4 ± 0.17	0.5 ± 0.1	0.74 ± 0.26	0.45 ± 0.17

* Data from Malakpour Kolbadinezhad et al. [24].

Muscle water content was significantly lower in SW-L (48h) acclimated fishes indicating dehydration but was unaffected by BW acclimation or BW-L ligation (BW-L). Muscle Na^+^ and K^+^ concentrations were significantly higher in SW ligated fish, although with no differences in Na^+^: K^+^ ratio in muscle.

### NKA activity

**Fig 1:**
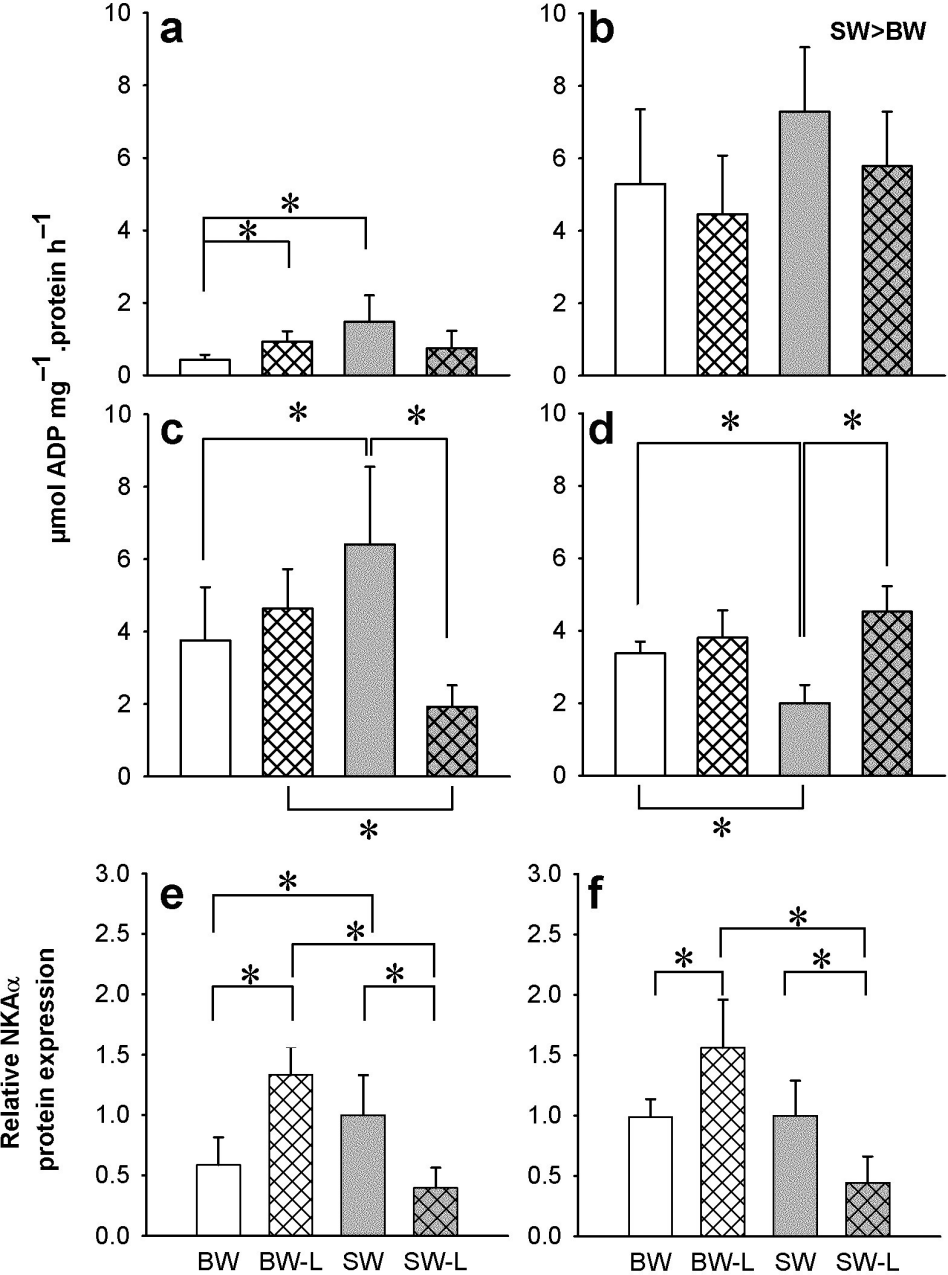
Osmoregulatory organ Na^+^/K^+^-ATPase activity and protein expression. NKA activity in the gill (**a**), kidney (**b**), anterior and and posterior intestine (**c**-**d**, respectively) of *P*. *lineatus* acclimated to [brackish water (BW), brackish water ligated (BW-L) 3‰, seawater (SW), and seawater ligated (SW-L) 34‰] conditions. Relative expression of NKA α subunit protein in the anterior and posterior intestine are presented in (**e-f**). Values are means ± S.D. (n = 5–6). The asterisks indicate a significant difference between the groups where there was an interaction of salinity and ligation, two-way ANOVA and SNK. Differences between BW and SW are also indicated. (*P < 0*.*05*). Sham non-ligated data are from Malakpour Kolbadinezhad et al. [24].

In SW acclimated catfish, specific NKA activity was lowest in gill and posterior intestine, and more than three times higher in kidney and anterior intestine (Fig 1). In response to SW-L no significant differences in NKA activity were detected in gill, or kidney (Fig 1A and 1B). However, in anterior and posterior intestine NKA activity was 2x lower and 2x higher, respectively than their respective SW sham controls (Fig 1C and 1D). Kidney showed significantly higher NKA activity in SW versus BW acclimated fish, but no ligation effect (Fig 1B). In BW-L NKA activity only increased in the gill while sham BW gill was also lower than the corresponding SW sham group (Fig 1A).

### Western blotting

We used antibodies cross reactive with NKA α-subunits, NKCC, cytosolic carbonic anhydrase, VHA B subunit, Slc26a6 and heat shock protein (Hsp70) to determine how DO ligation and salinity affected the abundance of these important ion transporters in gill, kidney, anterior and posterior intestine. We were unable to detect Cftr, Nkcc, or Slc26a6 [8] in these tissues using heterologous antibodies, thus no results are shown for these transporters (also see Malakpour Kolbadinezhad et al. [24]).

NKA α-subunit expression was detected in all organs of interest as a single band of approximately 100kDa (S1A Fig). The relative expression of the NKA α-subunit protein was not salinity responsive in gill and kidney (data not shown), but in anterior and posterior intestine expression levels were dependent on both salinity and ligation (S1E and S1F Fig). In anterior intestine, NKA α-subunit expression was significantly lower in BW acclimated fish compared to SW reflecting differences in NKA activity. In SW acclimated fish, both intestinal regions had significantly lower protein expression following ligation. However, this was only consistent with NKA activity in the anterior intestine, while the opposite was observed in posterior intestine. Conversely, BW ligation resulted in higher protein expression in both regions; however, there were no differences in NKA activity. In both regions, the highest levels of NKA protein expression were present in BW ligated fish. NKCC (T4) expression was not detected in any of the tissues in the current experiment (data not shown).

**Fig 2:**
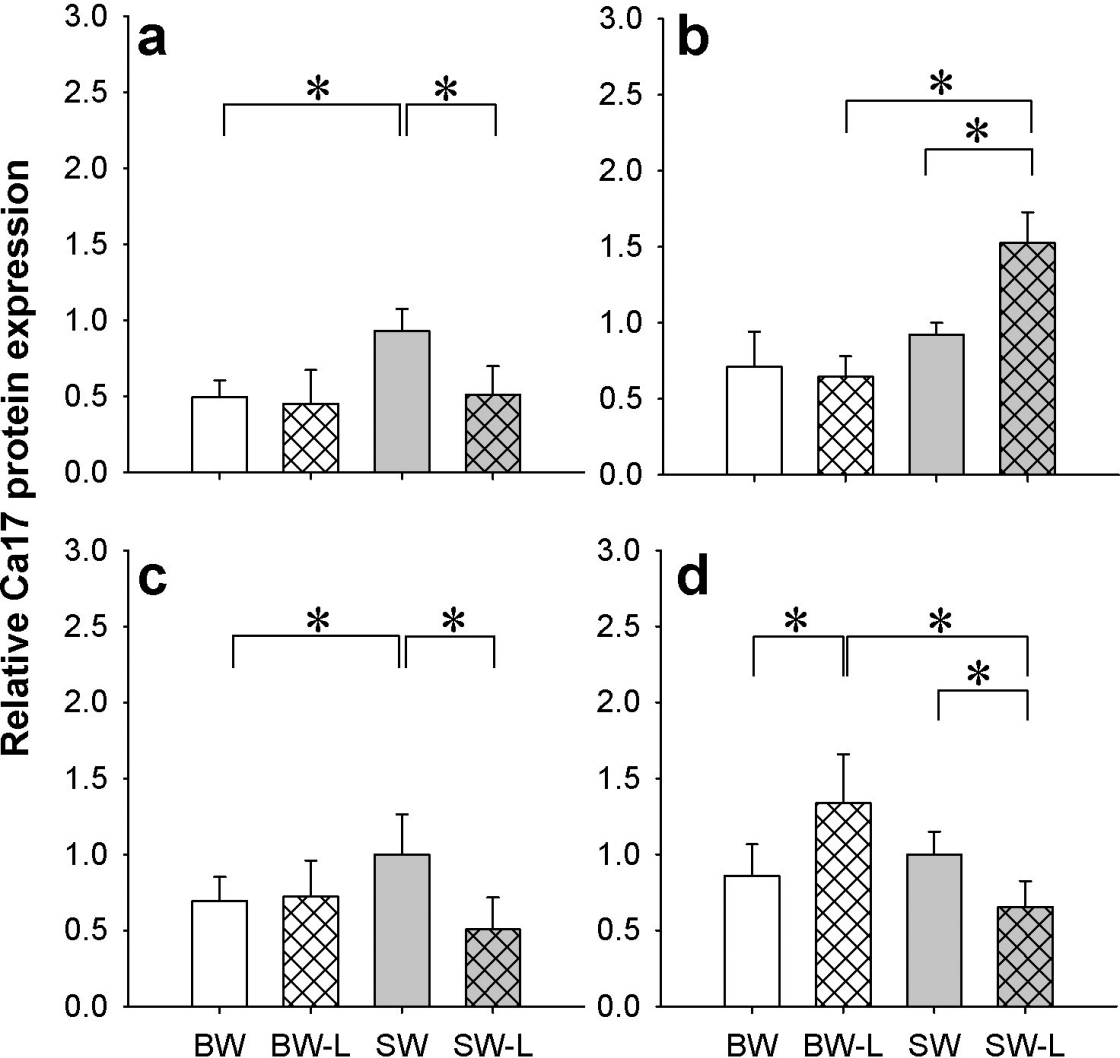
Osmoregulatory organ carbonic anhydrase protein levels. Relative expression of cytosolic carbonic anhydrase (CA) protein in the gill (**a**), kidney (**b**), anterior and posterior intestine (**c-d**, respectively) of *P*. *lineatus* acclimated to [brackish water (BW), brackish water ligated (BW-L) 3‰, seawater (SW), and seawater ligated (SW-L) 34‰] conditions. Values are presented as means ± S.D of protein abundance (n = 5–6). The asterisks indicate a significant difference between the groups where there was an interaction of salinity and ligation, two-way ANOVA and SNK. Sham non-ligated data are from Malakpour Kolbadinezhad et al. [24].

Ca17-like immunoreactivity was detected as an approximately 30kDa band (S1C Fig) and interactions between salinity and ligation were detected in all tissues examined (Fig 2). In gill and anterior intestine Ca17 was lower in BW versus SW sham fish but no differences were detected in kidney or posterior intestine. Ligation in SW fish resulted in lower Ca17 levels in gill, anterior and posterior intestine (Fig 2A, 2C and 2D) while higher expression was found in kidney (Fig 2B). With BW ligation, significantly higher Ca17 was found only in posterior intestine relative to the BW sham group but no differences were observed in the other tissues (Fig 2D).

**Fig 3:**
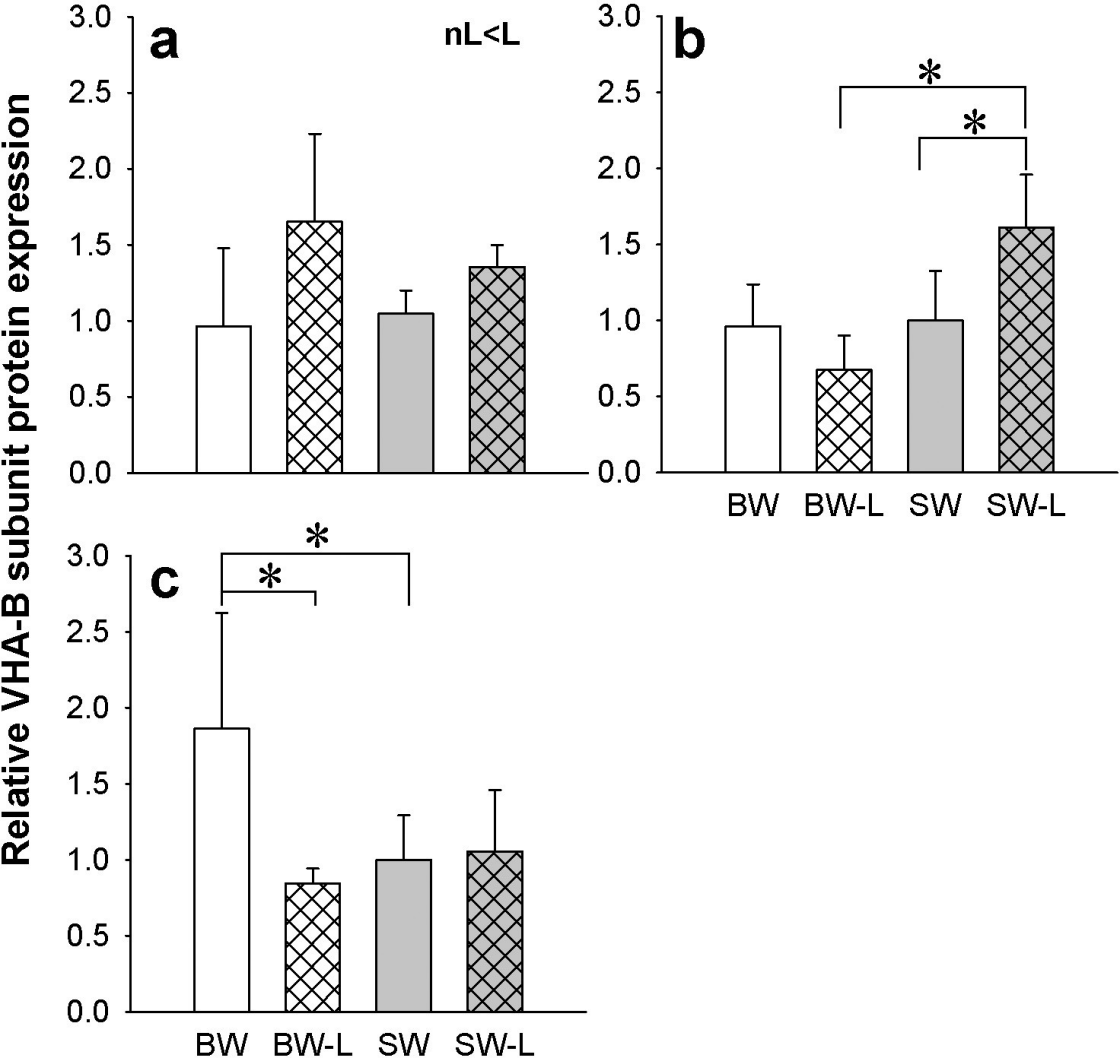
Osmoregulatory organ VHA protein levels. Relative western blotting expression of VHA B subunit in the gill (**a**), kidney (**b**) and posterior intestine (**c**) of *P*. *lineatus* acclimated to [brackish water (BW), brackish water ligated (BW-L) 3‰, seawater (SW), and seawater ligated (SW-L) 34‰] conditions. Values are presented as means ± S.D of protein abundance (n = 5–6). The asterisks indicate significant differences between the groups where there was an interaction of salinity and ligation, two-way ANOVA and SNK. Differences between non-ligation (nL) and ligation (L) are also indicated. (*P < 0*.*05*). Sham non-ligated data are from Malakpour Kolbadinezhad et al. [24].

The VHA B subunit (VHAB) was found expressed as a ~56 kDa band (S1B Fig) in gill, kidney and posterior intestine (Fig 3) but not anterior intestine (data not shown). In gill, VHAB levels were significantly higher in ligated versus non-ligated fish irrespective of acclimation salinity (Fig 3A). In kidney and posterior intestine there were interactions between salinity and ligation. In kidney, ligation resulted in higher VHAB compared to both SW-control and BW-L (Fig 3B). In posterior intestine, the BW sham group had higher VHAB compared to both BW-L and SW controls (Fig 3C).

**Fig 4:**
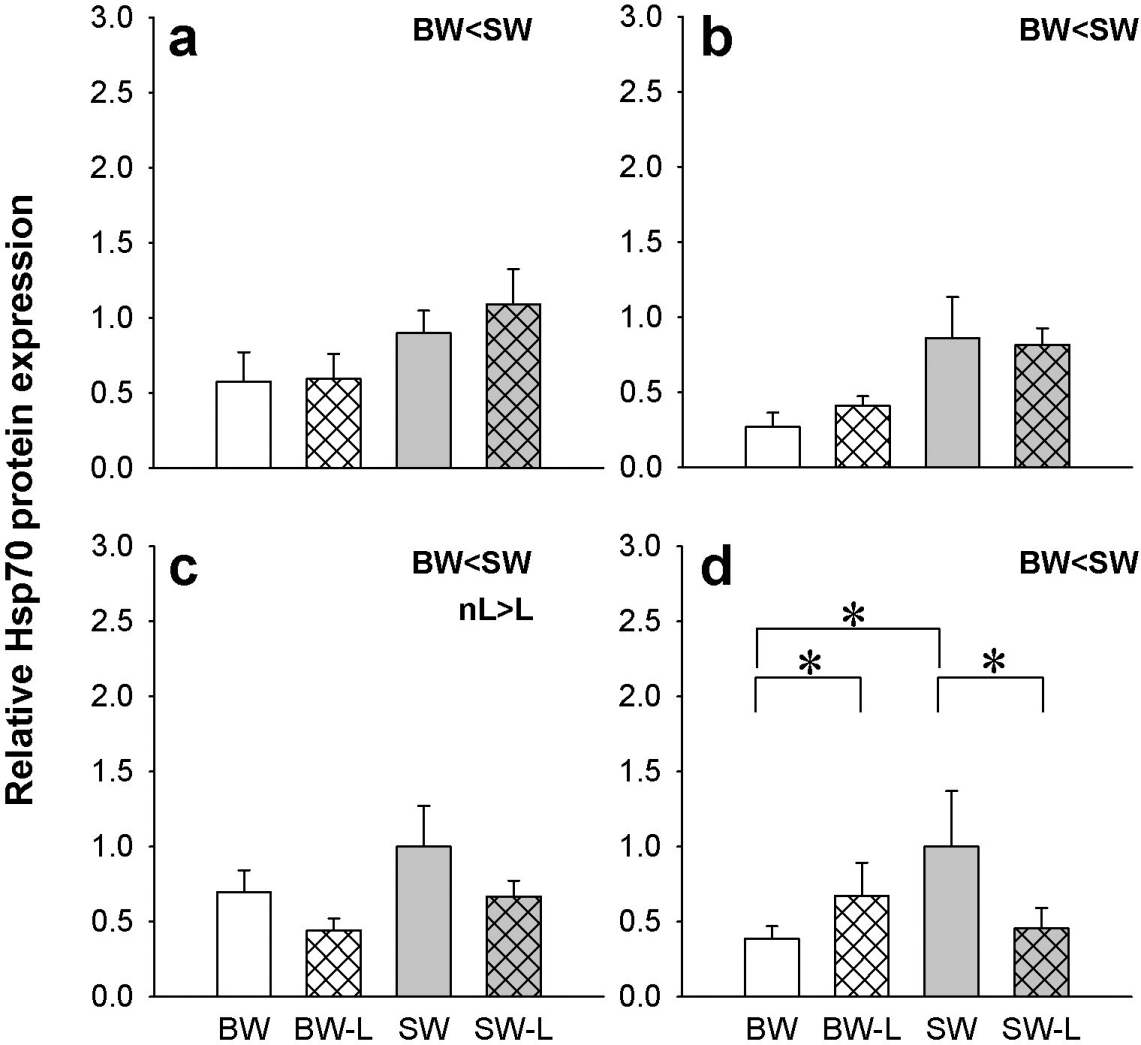
Osmoregulatory organ Hsp70 levels. Relative expression of heat shock protein 70 (Hsp70) in the gill (**a**), kidney (**b**), anterior and posterior intestine (**c,d**) of *P*. *lineatus* acclimated to [brackish water (BW), brackish water ligated (BW-L) 3‰, seawater (SW), and seawater ligated (SW-L) 34‰] conditions. Values are presented as means ± S.D of protein abundance (n = 5–6). The asterisks indicate a significant difference between the groups where there was an interaction of salinity and ligation, two-way ANOVA and SNK. Differences between BW and SW, and non-ligation (nL) and ligation (L) are also indicated. (*P < 0*.*05*). Sham non-ligated data are from Malakpour Kolbadinezhad et al. [24].

Hsp70 protein was detected in all of the tissues of interest in our work as a single 70 kDa immunoreactive band (S1D Fig). In all tissues Hsp70 levels were higher in SW versus BW fish. However, in anterior intestine ligation was associated with significantly lower Hsp70 levels independent of salinity (Fig 4C). An interaction between salinity and ligation was only found in the posterior intestine (Fig 4D). Ligation in SW fish was associated with lower Hsp70 levels whereas the opposite was observed in BW with significantly higher ligation associated expression. SW sham control Hsp70 levels were higher than respective BW shams.

### Gene expression of atp1a1, ca17, cftr, slc26a6a

**Fig 5:**
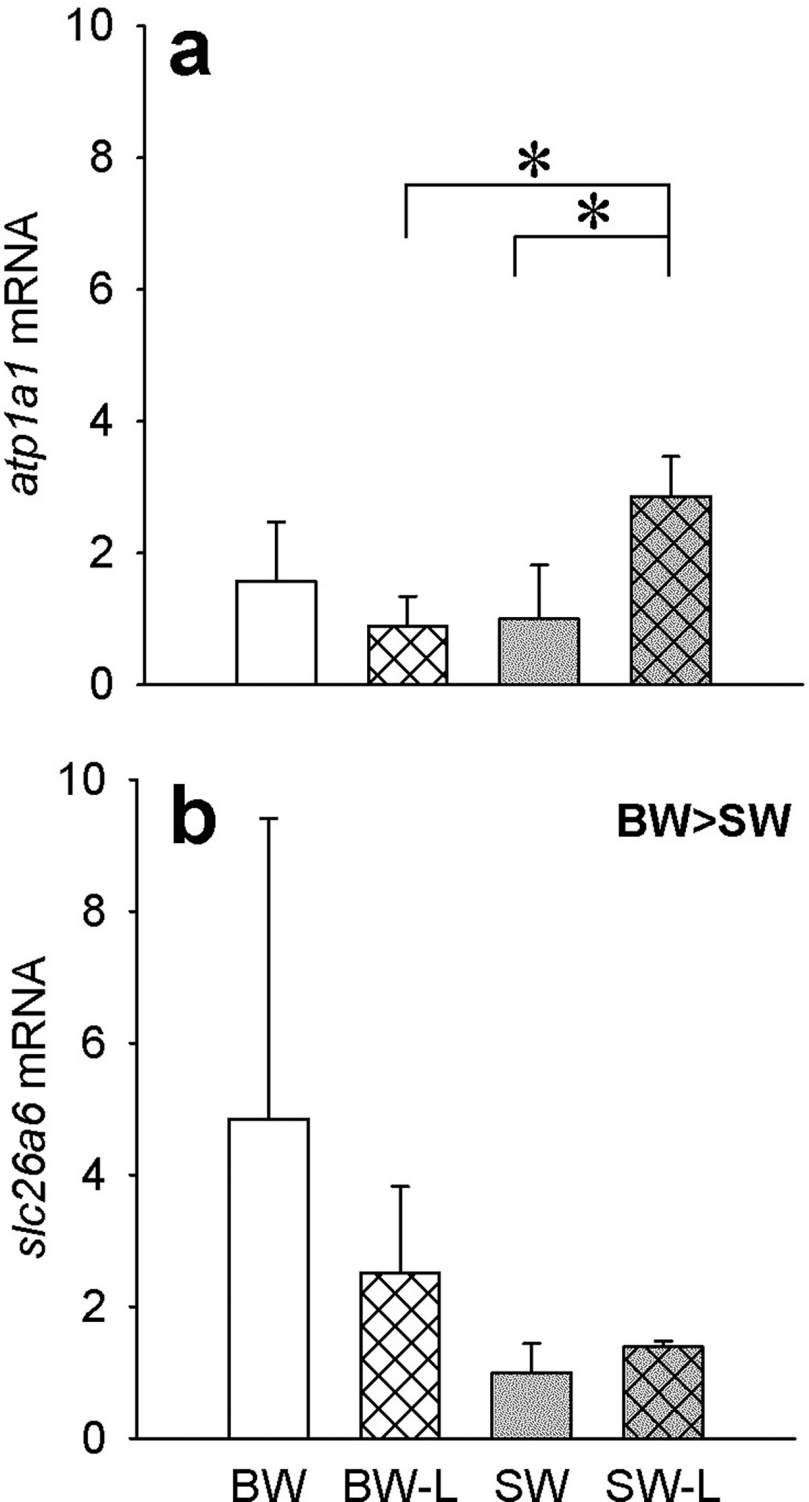
Osmoregulatory organ mRNA expression levels. Relative mRNA expression of kidney *atp1a1* (**a**) and *slc26a6* (**b**) in *P*. *lineatus* acclimated to [brackish water (BW), brackish water ligated (BW-L) 3‰, seawater (SW), and seawater ligated (SW-L) 34‰] conditions. Data are means ± S.D. (n = 3–4). The mRNA expression was normalized to the corresponding *bactin* abundance from the same sample and the expressed relative to the SW-control. The asterisks indicate a significant difference between the groups where there was an interaction of salinity and ligation, two-way ANOVA and SNK. Sham non-ligated data are from Malakpour Kolbadinezhad et al. [24].

NKA α-subunit *atp1a1* mRNA expression levels were measured in all organs but differences were only found in kidney. Ligation in SW fish was associated with significantly higher expression levels compared to SW controls and BW-L. (Fig 5A). Although detected in all organs, no differences in *cftr* or *ca17* mRNA expression were observed with either salinity or ligation (data not shown). The *slc26a6a* mRNA levels were found expressed in all tissues studied. However, only in kidney was a salinity effect observed. BW fish (sham and ligated) had higher mRNA levels compared to SW (sham and ligated) (Fig 5B).

### Immunohistochemistry

IHC staining pattern of BW-L and SW-L fish gill and intestine were similar to the BW and SW-control acclimated fish; however, a few changes were noted which are presented in the following section.

#### Gill

**Fig 6:**
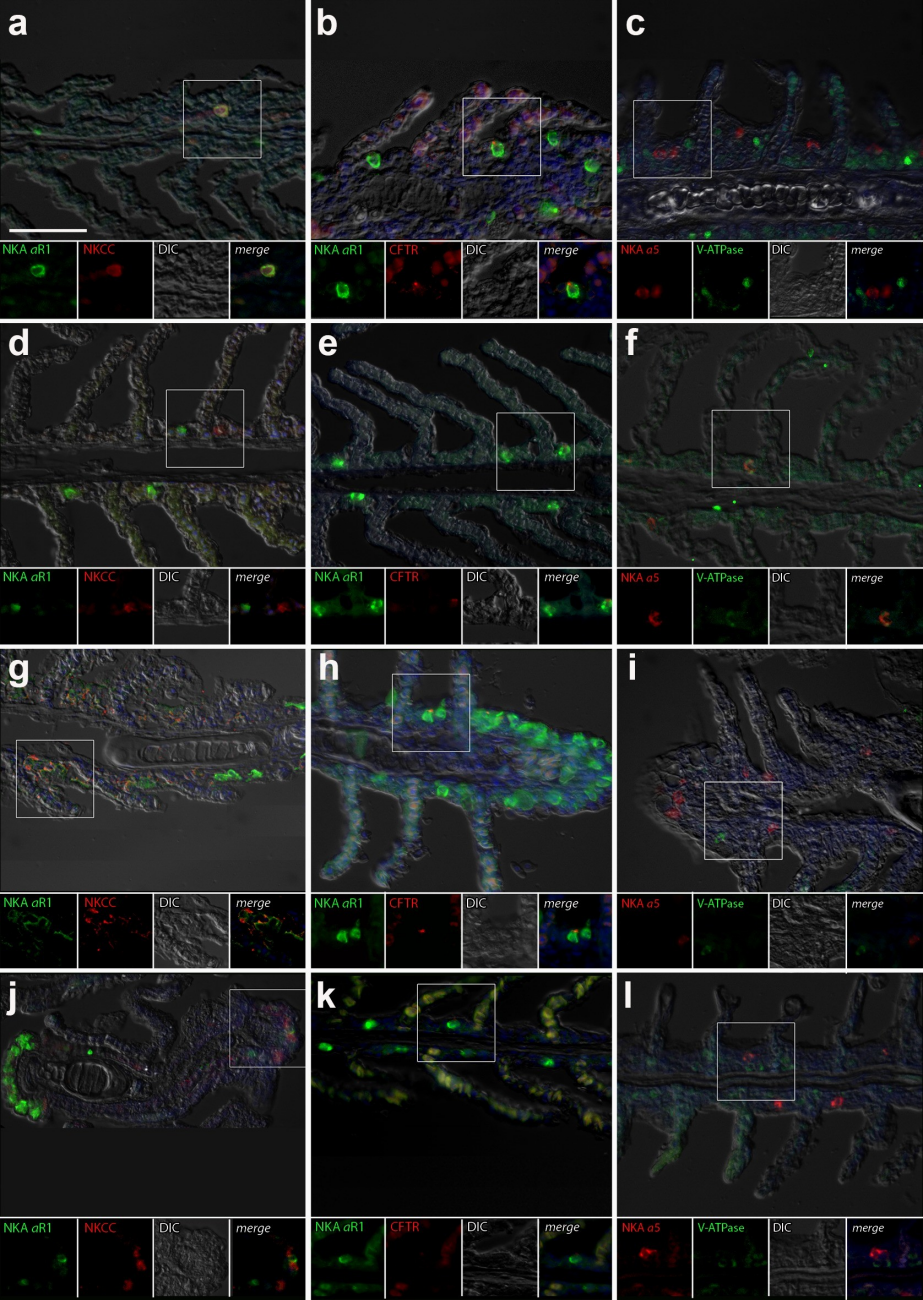
Gill immunohistochemistry. Double immunofluorescence localization of NKA (αR1, green **a, b, d, e, g, h, j, k**) with NKCC1 (T4, red **a, d, g, j**) and CFTR (red, **b, e, h, k**) or NKA (α5, red **c, f, i, l**) with VHA (B2, green **c, f, i**). Sections were counter stained with DAPI nuclear stain (blue) and overlaid with the differential interference contrast (DIC) images in the gills of *P*. *lineatus* acclimated in brackish water (BW) 3‰ (**a-c**), brackish water ligated (BW-L) 3‰ (**d-f**), seawater (SW-control) 34‰ (**g-i**) and seawater (SW-control) ligated (SW-C) 34‰ (**j-l**). Scale bar 100 µm in upper panel.

The gills of *P*. *lineatus* have a typical teleost gill organization of filaments with lamellae. In the branchial epithelium strong NKA immunoreactivity (IR) was detected in large isolated ovoid cells throughout the cytoplasm with the exception of the apical region (Fig 6). This NKA cellular staining pattern is typical of teleost fish chloride cell or ionocyte tubular system. There were relatively few of these branchial NKA-IR cells which were present in a heterogeneous distribution limited to a few interlamellar regions over the leading edge of the filament and were absent from the lamella. Experimental salinities and ligation did not alter the NKA-IR cell distribution pattern. The secretory Na^+^:K^+^:2Cl^−^cotransporter (NKCC1) expression in gill was rarely detected despite the use of antigen retrieval techniques and positive immunoreactivity in other tissues (kidney and intestine) indicating that species specific immunoreactivity problems were not an issue. The colocalization of NKCC1 in more weakly NKA-IR cells in BW and SW fish are shown in Fig 6A and 6G. Ovoid cells in the filament epithelium showing only NKCC1 staining were observed in BW-L and SW-L (Fig 6D, j) as well as SW control fish. The apical localization of CFTR was detected in some branchial NKA-IR cells with no apparent salinity and/or ligation dependent differences (Fig 6B, 6E, 6H and 6K). Some diffuse staining in red blood cell was also observed. Under all experimental conditions the VHA was localized in a similar cytoplasmic staining pattern as NKA; although in cells not showing NKA immunoreactivity and thus a distinct cell type(Fig 6C, 6I and 6L). However, colocalization of VHA with NKA-IR ionocytes was observed albeit rarely in BW-L fish (Fig 6F).

#### Intestine

**Fig 7:**
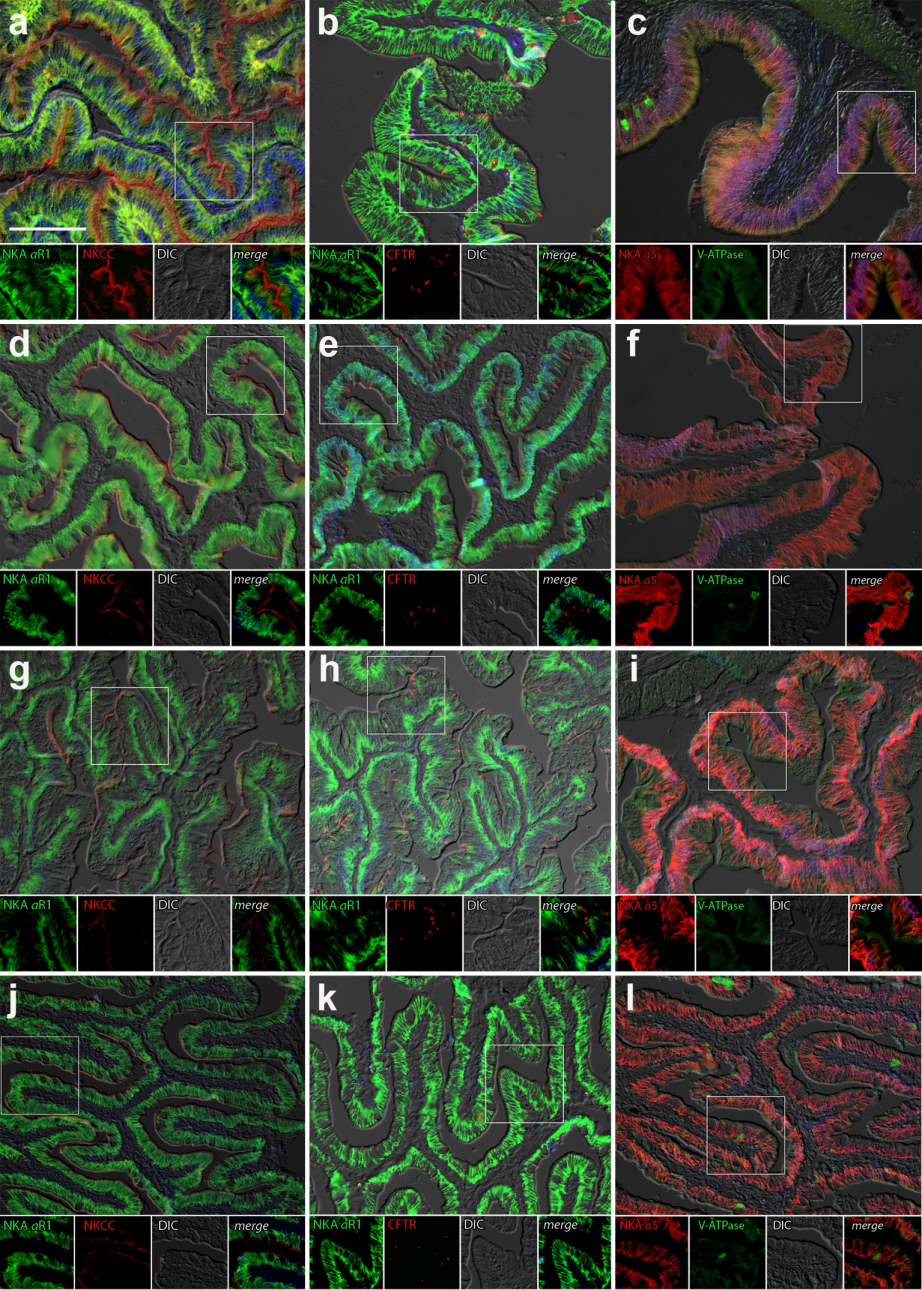
Anterior intestine immunohistochemistry. Double immunofluorescence localization of NKA (αR1, green **a, b, d, e, g, h, j, k**) with NKCC1 (T4, red **a, d, g, j**) and CFTR (red, **b, e, h, k**) or NKA (α5, red **c, f, i, l**) with VHA (B2, green **c, f, i**). Sections were counter stained with DAPI nuclear stain (blue) and overlaid with the differential interference contrast (DIC) images in the anterior intestine of marine catfish *P*. *lineatus* acclimated in brackish water (BW) 3‰ (**a-c**), brackish water ligated (BW-L) 3‰ (**d-f**), seawater (SW-control) 34‰ (**g-i**) and seawater (SW-control) ligated (SW-C) 34‰ (**j-l**). Scale bar 100 µm in upper panel.

**Fig 8:**
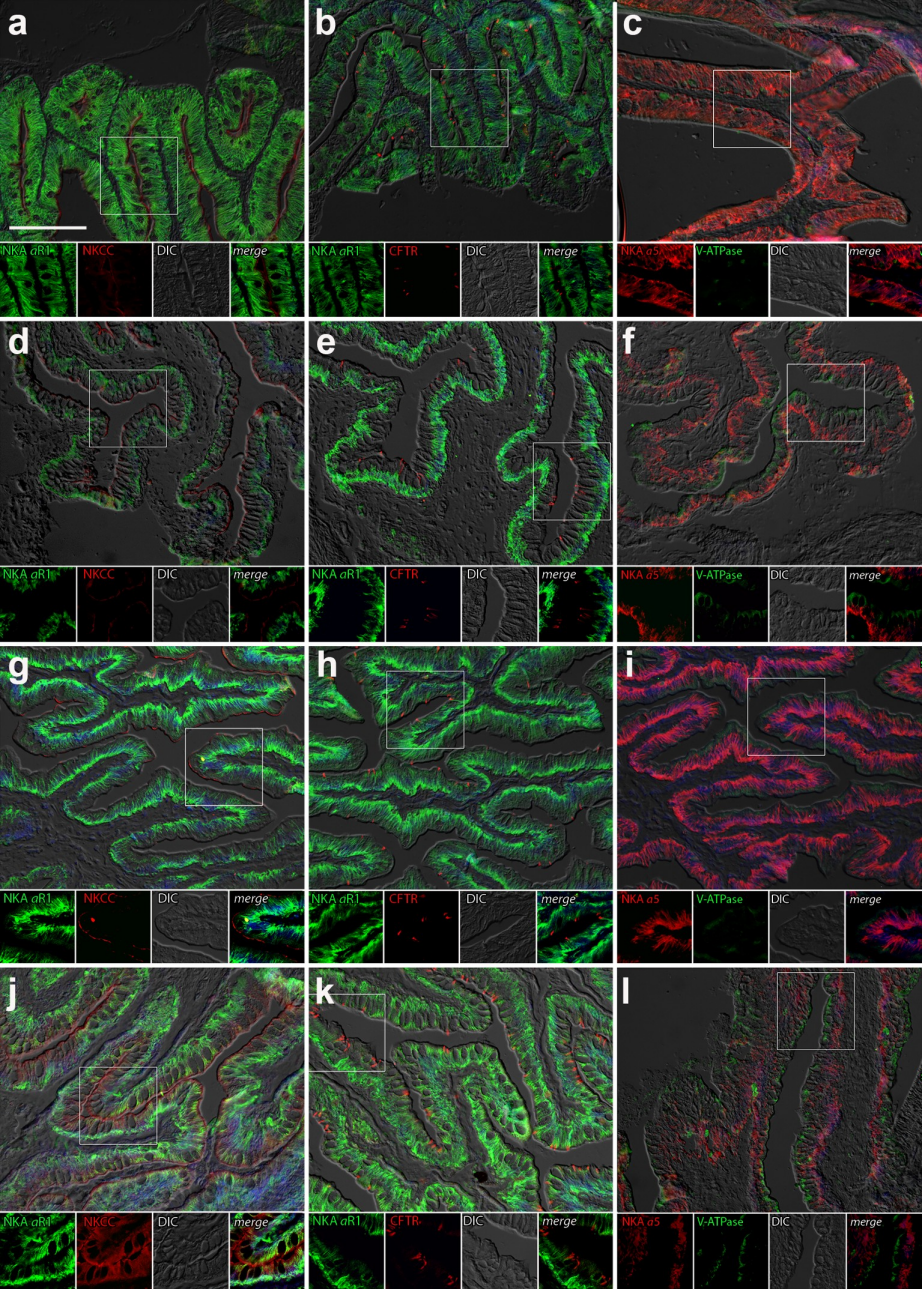
Posterior intestine immunohistochemistry. Double immunofluorescence localization of NKA (αR1, green **a, b, d, e, g, h, j, k**) with NKCC1 (T4, red **a, d, g, j**) and CFTR (red, **b, e, h, k**) or NKA (α5, red **c, f, i, l**) with VHA (B2, green **c, f, i**). Sections were counter stained with DAPI nuclear stain (blue) and overlaid with the differential interference contrast (DIC) images in the posterior intestine of *P*. *lineatus* acclimated in brackish water (BW) 3‰ (**a-c**), brackish water ligated (BW-L) 3‰ (**d-f**), seawater (SW-control) 34‰ (**g-i**) and seawater (SW-control) ligated (SW-C) 34‰ (**j-l**). Scale bar 100 µm in upper panel.

Immunolabeling of NKA in the anterior and posterior intestine of *P*. *lineatus* acclimated to BW or SW with or without DO ligation revealed intense staining in the basolateral regions of the intestinal epithelium (Figs 7 and 8). However, in posterior intestine of BW-L fish less staining compared to other groups of fish was observed (Fig 8D and 8E). NKCC2 or NCC immunoreactivity was detected in apical brush border of the epithelium in both the anterior and posterior intestine in all salinity experiments. However, basal staining in ligated SW fish posterior intestine of fish was also observed (Fig 8j). CFTR immunoreactivity was detected apically in isolated spindle shaped columnar cells in the epithelium of anterior and posterior intestine in all experimental groups (Fig 8B, 8E, 8H and 8K). However, in posterior intestine the frequency of these cells in SW ligated fish appeared greater (Fig 8K). There was a diffuse apical or subapical cytoplasmic localization of VHA in columnar epithelial cells of SW ligated fish in the anterior intestine (Fig 7). Staining was not observed in the brush border and occasionally material in the lamina propria or basal part of the epithelium was immunoreactive (Fig 7C and 7L). In the posterior intestine, a similar diffuse apical expression of VHA was observed in SW and BW ligated and SW control fish (Fig 8J, 8D and 8G) but not in BW sham fish (Fig 8C).

## Discussion

This work together with the companion study by MalakpourKobadinezhad et al. (2018) demonstrate that the DO is central to successful osmoregulation in the marine Plotosidae catfish. Ligation was a simple and effective means of “knocking out” DO function which resulted in an osmoregulatory impairment and an inability of other osmoregulatory organs to compensate for DO loss. Significantly, we found that gill ionocytes did not express the suite of ion transporters for secondary active Cl^-^ secretion, which is typical in other marine teleosts. The successful independent invasion of marine environments by the Plotosid catfishes is clearly linked to the DO that is unique to this family of Siluriformes, an order that is otherwise almost exclusively found in fresh water.

### Osmoregulatory indicators

The plasma osmolalities of euryhaline species with marine and freshwater origins vary between 160–410 and 235–414 mOsm·kg^-1^ H_2_O, respectively [44]. The observed plasma ion concentrations of *P*. *lineatus* were in the range of other teleost fish species (see review by [7]). Ligation of SW fish increased plasma Na^+^ and Cl^-^ ion concentrations, and the increased hematocrit and decreased muscle water content indicated a clear dehydration and osmoregulatory disturbance. There was also a transient increase in muscle Na^+^ concentration that recovered by 48h. A similar salt load buffering by muscle has also been reported in rectal gland ectomised lip shark (*Hemiscyllium plagiosum*) by Chan et al. [45]. A slightly higher although statistically insignificant muscle Na^+^ load in *S*. *acanthias* has been also been reported by Wilson et al [46]. In our work, SW ligation also caused a decrease in survival similar to Kowarsky [19] working with *C*. *macrocephalus*. The ligation or excision of the salt secreting rectal gland in elasmobranchs for prolonged periods (1 month) showed no significant effect on plasma electrolytes compared to controls in SW [46,47,48,49] or dilute seawater [47,50]. These results indicate that elasmobranch fishes are capable of a significant compensatory response, which is most likely renal [47]. Wilson et al. [46] could find no changes in gill ionocytes to suggest a branchial contribution to the response. The similarities of the Plotosid catfish DO and elasmobranch rectal gland in salt secretion are clear [17, 18, 23] since the molecular secondary active Cl^-^ secretory mechanisms are conserved [24].

Stress proteins (Hsps) have a vital role in cellular homeostatic processes [51, 52, 53]. In sea bream, Deane and Woo [25] have demonstrated that salinity stress elicits an Hsp70 response indicating a cellular stress. In *P*. *lineatus*, acclimation to hypersaline conditions (60‰) resulted in a significant increase in DO Hsp70 levels indicating a cellular stress response. In general, in the present study Hsp70 levels were salinity dependent but ligation had no effect on Hsp70 abundance.

### Brackish water challenge and ligation

In contrast to ligation of SW *P*. *lineatus*, there were no significant changes in osmoregulatory indicators in plasma or muscle in BW ligated fish compared to BW sham fish. The lack of changes is consistent with the premise that the DO is functioning in salt secretion. The 3‰ brackish water conditions are well below the iso-osmotic and iso-ionic levels that would necessitate salt secretion. Plasma Na^+^ levels were clearly lower in BW versus SW acclimated fishes indicating a shift in the osmoregulatory set point, which is not uncommon in euryhaline fishes [54]. In *C*. *macrocephalus* BW ligated fish showed a depression in plasma Na^+^ levels although no significant change in osmolality were observed [19] indicating a similar response.

Changes in the strong ion (Na^+^/Cl^-^) ratio (SIR) of plasma has been recommended for indicating acid-base imbalances [55, 56]. Since the direct measurements of plasma acid-base balance were not done in the current study due to the small size of the fish we instead calculated SIR which might reveal changes in the plasma levels of weak anions (e.g. HCO_3_^-^) and thus acid-base balance. However, although SIR was lower in BW compared to SW acclimated fish suggesting an acidosis [56], no effects of ligation were observed suggesting the absence of a ligation related acid-base disturbance.

### Evidence for role of gills in salt secretion?

In an earlier study in which *P*. *lineatus* was acclimated to a range of salinities (3–60‰), branchial NKA activity and gill ionocytes did not show a correlation with salinity which contrasted with the DO [24]. Significantly, gill ionocytes lacked strong basolateral NKA, and NKCC1 expression with apical CFTR staining which again could be contrasted with DO parenchymal cells. Taken together, these results in *P*. *lineatus* indicated that the DO is the main organ for ion regulation and not the gills, similar to the relationship between the rectal gland and gills in the elasmobranches [46, 57, 58]. Nonetheless, NKA, NKCC1 and CFTR are all still found in the gill although not collocalized in the same cell type. Even though we were unable to confirm tissue expression of CFTR and NKCC1 by Western blotting, we are confident of the specific crossreactivity of the antibodies used [CFTR c-term (R&D systems) and T4 clone (DSHB), respectively], since they have been used in numerous other studies in teleost fishes (reviewed by [1, 59, 60]). The staining pattern of the T4 antibody indicates NKCC1 immunoreactivty based on the cytosolic staining pattern. As noted earlier the T4 antibody also crossreacts with NCC and NKCC2 but these can be distinguished based on their apical distribution [60,61]. The sparsity of immunoreactive cells indicates that the tissue protein expression levels were likely well below the sensitivity of the Western blotting technique. As a corollary, in our companion study MalakpourKobadinezhad et al. [24] we were able to demonstrate appropriate antibody crossreactivity in Western blots of dendritic organ that presumably presents higher expression levels.

The question thus arises, “During dendritic organ ligation are gill ionocytes mobilized to perform salt secretion?” In ligated SW fish, we could find no evidence of increased NKA, NKCC or CFTR expression at either the mRNA, or protein levels, and there were no cellular level changes in distribution that would have been consistent with a compensatory response by the gills. In addition, carbonic anhydrase protein levels decreased which is significant because carbonic anhydrase catalyzes the reversible dehydration/hydration reactions of CO_2_ and has an obvious role in ion and acid-base regulation [1, 14]. The DO ligation results are consistent with findings in marine elasmobranchs where removal of rectal gland resulted in no significant change in branchial NKA activity, ionocytes number or ultrastructure in spiny dogfish [46].

In contrast to ligation in SW fish, NKA activity levels were significantly elevated in BW ligated animals compared to BW controls. However, we observed discrepancies between NKA activity and α subunit protein and mRNA (*atp1a1*) expression in gills, which did not change, suggesting that there might be post translational and/or post-transcriptional modification. Under the hypo-ionic BW conditions, branchial ionocytes may be mobilized to compensate for lost ion uptake by the DO. In support, VHAB protein levels were significantly higher in ligated animals. The VHA has been shown to be involved in acid-base and/or ion regulation [1]. IHC results indicate that VHA was present in a subpopulation of ionocytes in the filament epithelium with a lack of consistent colocalization with NKA-IR cells. This pattern has been reported in a number of different species [62, 63, 64].

### Response in intestine and kidney

In marine fishes, the intestine has an important role in osmoregulation through absorption of imbibed water to compensate for the passive loss of water across body surfaces by osmosis (see reviews by [6, 7]). Water uptake is driven by solute-linked water transport whereby localized osmotic gradients are generated by active solute transport. Ion transport involves apical NCC/NKCC and basolateral NKA, which have been found expressed in *P*. *lineatus*. Significantly, the gill or DO would excrete the excess NaCl load associated with water uptake. Higher NKA activity in anterior intestine compared to posterior in SW-controls is consistent with greater water uptake rates observed in this region (see reviews by [6, 7]). The detection of VHAB in the posterior and not the anterior intestine by Western blotting is also consistent with VHA’s role in aiding the Cl^-^/HCO_3_^-^ exchanger’s role in water uptake in this region [9].

Although SW ligated fish show clear signs of dehydration and ion regulatory problems, we would hypothesize that drinking and water absorption might be inhibited since in the absence of the DO, continued drinking would only add to the NaCl load. In support, NKA α subunit level and cytosolic carbonic anhydrase protein levels (Ca17) in both intestinal regions decrease with ligation, which suggest a possible decreased transport role of the intestine [65]. In marine fishes, HCO_3_^-^ secretion has been linked to enhanced water uptake involving cytosolic CA, and apical VHA and Slc26a6 Cl^-^/HCO_3_^-^ exchanger. The changes following ligation indicate a complex response, which will require measurements of drink rates and water flux rates to address.

Based on our observations of ion transporter distribution patterns in posterior intestine, we hypothesize that the posterior intestine takes on a salt secretory function to contribute to osmoregulation in the absence of the DO and a lack of a response from the gills. Salt secretion has been shown to occur in fed Gulf killifish posterior intestine [10], requiring the apical expression of CFTR and basolateral expression of NKA and NKCC1. Indeed, in SW ligated *P*. *lineatus* apical CFTR staining cells are more common and NKCC1 staining is observed. However, NKA activity and α subunit level decrease with ligation in SW, and no changes in *cftr* mRNA levels were found. It was also not possible to confirm these finding by Western blotting for Cftr or NKCC1 in intestine so caution should be used when evaluating these findings. This somewhat unorthodox switching of roles of the posterior intestine is not without precedence in fishes. In male freshwater stickleback (*Gasterosteus aculeatus*), the kidney takes on the role of secreting an adhesive substance for nest building during the breeding season, and the posterior intestine takes of the task of dilute fluid formation for osmoregulation [66, 67].

The biological significance of the higher intestinal NKA activity in BW ligated fish compared to shams is unclear given that drinking and water absorption are unlikely under these conditions (see reviews by [6, 7]). In addition, the posterior intestine also showed higher protein expression of Ca17 and Hsp70 suggesting that ligation is altering intestinal physiology.

The kidney in SW fish has a reduced glomerular filtration rate and urine production rate in order to conserve water. In marine teleost fishes, urine is typically iso-osmotic with relatively high concentrations of divalent ions. However, the Plotosid catfish *C*. *macrocephalus* [19] has been reported to produce hyper-osmotic urine. *P*. *lineatus* is unusual in having higher renal NKA activity in BW versus SW, which is the opposite of what is observed in other euryhaline fishes but would be consistent with hyper-osmotic urine production [24]. Ligation, however, did not alter NKA activity or protein levels suggesting no compensatory response of the kidney although mRNA levels did. This contrasts with rectal glandectomy in elasmobranchs where a renal compensation has been observed [47]. Although NKA did not increase, VHA and CA protein levels both did. Renal CA and VHA both have a significant role in HCO_3_^–^ reabsorption and urine acidification [14, 68]. Therefore, it seems that ligated SW fish may be experiencing an acid-base imbalance that is being compensated for by the kidney. Successful compensation of the suspected acid-base imbalance is supported by the unchanged plasma strong ion ratio.

## Conclusions

Since the DO is an external organ, ligation was an effective, non-invasive approach to examine loss of function. It provided valuable evidence about the importance of the DO as well as the compensatory responses of the other osmoregulatory organs (gill, kidney and intestine). We found that the ligation of SW acclimated fish increased ions and osmolality of plasma while it had negative effects on survival and generally osmoregulatory capacity of fish. Notably fish could not survive without the DO in HSW. From the loss of the DO through ligation, a compensatory response from the other osmoregulatory organs was predicted. However, ligation in SW did not alter gill or kidney NKA expression while, a decrease and increase were observed in anterior or posterior intestine, respectively. The DO was central to hypo-osmoregulation in the marine Plotosid catfish and gill, kidney and intestine had a limited compensatory role. In general, the intestine was also the most responsive osmoregulatory organ in terms of ion transporter expression, but the patterns of change indicated a complex response that will require additional work in order for the changes to be properly interpreted. Thus, the DO represents a unique adaptation to hypo-osmoregulation in the Plotosid catfishes that can be contrasted with other marine teleost groups, which rely on their gill ionocytes for active ion regulation.

## Supporting information

S1 Checklist Plos-one-humane-endpoints-checklist. (DOCX)

S1 Fig Representative western blots. The figure shows immunoreactive bands for (a) NKA α-subunit (~100kDa), (b) VHA B subunit (56kDa), Ca17 (30kDa), hsp70 (70 kDa) and α tubulin (50 kDa). Ladder 250, 150, 100, 75, 50, 37.5 25 kDa (All Blue Prestained Protein Standards, BioRad).
(DOCX)

S1 Table Primer nucleotide sequences and amplicon sizes for RT-PCR and qPCR. Primers used in the present study for RT-PCR and qPCR (*actb*, β-Actin; *atp1a1*, Na^+^/K^+^-ATPase; *cftr*, cystic fibrosis transmembrane conductance regulator; *ca17*, cytosolic carbonic anhydrase; *slc26a6*, Putative Anion Transporter Cl^-^/HCO_3_^-^ exchanger gene).
(DOCX)

S2 Table RT-PCR profiles. Tested genes include *actb*, β-Actin; *atp1a1*, Na^+^/K^+^-ATPase; *cftr*, cystic fibrosis transmembrane conductance regulator; *ca17*, Cytosolic carbonic anhydrase; *slc26a6*, Putative Anion Transporter Cl^-^/HCO_3_^-^ exchanger.
(DOCX)

S3 Table Real time RT-PCR conditions using iQ SYBR green supermix. actb, β-Actin; *atp1a1*, Na^+^/K^+^-ATPase; *cftr*, cystic fibrosis transmembrane conductance regulator; *ca17*, cytosolic carbonic anhydrase; *slc26a6*, Putative Anion Transporter Cl^-^/HCO_3_^-^ exchanger gene.
(DOCX)
