# Supplementary material for: Effect of dendritic organ ligation on striped eel catfish Plotosus lineatus osmoregulation
Source: PLoS One. 2018 Oct 23;13(10):e0206206. doi: 10.1371/journal.pone.0206206 (PMC6198982; doi:10.1371/journal.pone.0206206)
Supplement: S1 Fig — The figure shows immunoreactive bands for (a) NKA α-subunit (~100kDa), (b) VHA B subunit (56kDa), Ca17 (30kDa), hsp70 (70 kDa) and α tubulin (50 kDa). Ladder 250, 150, 100, 75, 50, 37.5 25 kDa (All Blue Prestained Protein Standards, BioRad). (DOCX) [file pone.0206206.s002.docx]

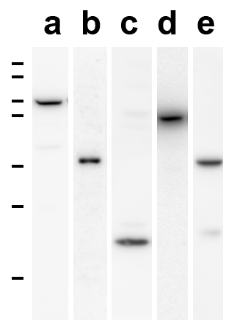


**Fig. S1. Representative western blot** showing immunoreactive bands for (a) NKA α-subunit (~100kDa), (b) VHA B subunit (56kDa), Ca17 (30kDa), hsp70 (70 kDa) and α tubulin (50 kDa). Ladder 250, 150, 100, 75, 50, 37.5 25 kDa (All Blue Prestained Protein Standards, BioRad).
