# Supplementary material for: Effect of dendritic organ ligation on striped eel catfish Plotosus lineatus osmoregulation
Source: PLoS One. 2018 Oct 23;13(10):e0206206. doi: 10.1371/journal.pone.0206206 (PMC6198982; doi:10.1371/journal.pone.0206206)
Supplement: S1 Table — Primers used in the present study for RT-PCR and qPCR (actb, β-Actin; atp1a1, Na+/K+-ATPase; cftr, cystic fibrosis transmembrane conductance regulator; ca17, cytosolic carbonic anhydrase; slc26a6, Putative Anion Transporter Cl-/HCO3- exchanger gene). (DOCX) [file pone.0206206.s003.docx]

**S1 Table.** Nucleotide sequences and amplicon sizes of primers used in the present study for RT-PCR and qPCR (*actb*, β-Actin; *atp1a1*, Na^+^/K^+^-ATPase; *cftr*, cystic fibrosis transmembrane conductance regulator; *ca17*, cytosolic carbonic anhydrase; *slc26a6*, Putative Anion Transporter Cl^-^/HCO_3_^-^ exchanger gene).

**RT-PCR Forward Reverse Size (bp)**

*actb* GGCCGCGACCTACAGACTAC ACCGAGGAAGGATGGCTGGAA 250

*atp1a* GGTATCGCTGGGTCTGATGT AGGATGGAGTTCCTCCTGGT 632

*cftr* TTTTCTGGTGGACAAGCCCT ATCGGGAAAGTGCTCGTAGG 188

*ca17* CAGTTCCATTTCCATTGGGG CAGAGGAGGGGTGGTCAG 333

*slc26a6* TGGTGCGGTTTGGATTTGTG ACCAGTTCCTGGTTGCTGTC 544

**qPCR**

*actb* CCCTCGTGCTGTGTTCCCATC CTCTTGCTCTGTGCCTCATCTCC 108

*atp1a* CCACTTTAGCCTCTCTGATGAC ATAACCTTGATTCCAGCACTCC 183

*cftr* TTTTCTGGTGGACAAGCCCT ATCGGGAAAGTGCTCGTAGG 188

*ca17* GGGGATCCAGTAATGAGAAGG CAAGAAGACCCCAACCACAG 152

*slc26a6* ATCAATCCGAACCGCTACAG AACAGGAATCGGAACAGGAA 183
