# Supplementary material for: Effect of dendritic organ ligation on striped eel catfish Plotosus lineatus osmoregulation
Source: PLoS One. 2018 Oct 23;13(10):e0206206. doi: 10.1371/journal.pone.0206206 (PMC6198982; doi:10.1371/journal.pone.0206206)
Supplement: S2 Table — Tested genes include actb, β-Actin; atp1a1, Na+/K+-ATPase; cftr, cystic fibrosis transmembrane conductance regulator; ca17, Cytosolic carbonic anhydrase; slc26a6, Putative Anion Transporter Cl-/HCO3- exchanger. (DOCX) [file pone.0206206.s004.docx]

**S2 Table.** RT-PCR profiles (*actb*, β-Actin; *atp1a1*, Na^+^/K^+^-ATPase; *cftr*, cystic fibrosis transmembrane conductance regulator; *ca17*, Cytosolic carbonic anhydrase; *slc26a6*, Putative Anion Transporter Cl^-^/HCO_3_^-^ exchanger)

| **Step** |  | **RT-PCR** | ***actb*** | ***atp1a*** | ***cftr*** | ***ca17*** | ***slc26a6*** |  |
| --- | --- | --- | --- | --- | --- | --- | --- | --- |
| **1** |  | **Denaturation**  **hot start** | 95°C  2min | 98°C  10s | 98°C  10s | 98°C  10s | 98°C  10s |  |
| **2** |  | **Denature** | 95°C  30s | 98°C  1s | 98°C  1s | 98°C  1s | 98°C  1s |  |
| **3** |  | **Anneal** | 60°C  30s | 60°C  5s | 58°C  5s | 56°C  5s | 58°C  5s |  |
| **4** |  | **Extend** | 72°C  5s | 72°C  5s | 72°C  5s | 72°C  3s | 72°C  3s |  |
| **5** |  | **Repeat 2-4** | 30 | 35 | 35 | 35 | 35 |  |
| **6** |  | **Final**  **Extend** | 72°C  5min | 72°C  2min | 72°C  2min | 72°C  2min | 72°C  2min |  |
