# Supplementary material for: Effect of dendritic organ ligation on striped eel catfish Plotosus lineatus osmoregulation
Source: PLoS One. 2018 Oct 23;13(10):e0206206. doi: 10.1371/journal.pone.0206206 (PMC6198982; doi:10.1371/journal.pone.0206206)
Supplement: S3 Table — actb, β-Actin; atp1a1, Na+/K+-ATPase; cftr, cystic fibrosis transmembrane conductance regulator; ca17, cytosolic carbonic anhydrase; slc26a6, Putative Anion Transporter Cl-/HCO3- exchanger gene. (DOCX) [file pone.0206206.s005.docx]

**S3 Table.** Real time RT-PCR conditions using iQ SYBR green supermix. actb, β-Actin; *atp1a1*, Na^+^/K^+^-ATPase; *cftr*, cystic fibrosis transmembrane conductance regulator; *ca17*, cytosolic carbonic anhydrase; *slc26a6*, Putative Anion Transporter Cl^-^/HCO_3_^-^ exchanger gene.

| **Step** | ***actb*** | ***atp1a*** | ***cftr*** | ***ca17*** | ***slc26a6*** |
| --- | --- | --- | --- | --- | --- |
| **Denaturation and hot start** | 95°C  3min | 95°C  3min | 95°C  3min | 95°C  3min | 95°C  3min |
| **Denaturation** | 95°C  10s | 95°C  10s | 95°C  10s | 95°C  10s | 95°C  10s |
| **Annealing** | 60°C  30s | 60°C  30s | 58°C  30s | 56°C  30s | 58°C  30s |
| **Extension** | 72°C  5s | 72°C  3s | 72°C  5s | 72°C  3s | 72°C  3s |
| **Cycle #** | 30 | 35 | 35 | 35 | 35 |
